# The incidental finding of xanthogranulomatous cholecystitis: a report of 10 cases

**DOI:** 10.1093/jscr/rjac443

**Published:** 2022-09-20

**Authors:** Abdulrahman Muaod Alotaibi, Eid Almasoudi, Hassan Ahmed, Abubakr Alzwaihiri

**Affiliations:** Department of Surgery, Faculty of Medicine, University of Jeddah, Jeddah, Saudi Arabia; Department of Surgery, Dr. Soliman Fakeeh Hospital, Jeddah, Saudi Arabia; Department of Surgery, Faculty of Medicine, University of Jeddah, Jeddah, Saudi Arabia; Department of Surgery, Faculty of Medicine, University of Jeddah, Jeddah, Saudi Arabia; Department of Surgery, Faculty of Medicine, University of Jeddah, Jeddah, Saudi Arabia

**Keywords:** xanthogranulomatous cholecystitis XGC, cholecystectomy, gallbladder disease

## Abstract

There is insufficient clinical knowledge about xanthogranulomatous cholecystitis (XGC) due to biased reporting. This study aims to investigate the incidence of XGC and evaluate the clinical outcome regarding operative time, rate of conversion and intraoperative or postoperative complications. We included 1141 patients who underwent surgery for gallbladder disease between January 2019 and December 2021. Of 1141 patients who underwent cholecystectomy, XGC was seen in 10 (0.87%). The average age is 47 (24–81 years old) with a male to female ratio of 3:2. Biliary pancreatitis and obstructive jaundice are seen in two patients who did ERCP and stenting before surgery. One patient presented with a 4 cm necrotizing soft-tissue granuloma. The BMI was high, with class I obesity in five patients. Symptoms last from 1 to 12 weeks. One patient was only diagnosed preoperatively as XGC. Four out of 10 (40%) required more than 72 h of hospitalization. All patients underwent elective sitting surgery, with eight successfully managed by laparoscopy and one converted to open. The average operative time was 90 min (43–193 min), and a postoperative drain was inserted in four patients. The median follow-up is after 24 months (11–30 months), with no postoperative collection, bleeding, complication or readmission. XGC is a rare benign entity requiring no further action upon incidental discovery. Surgical resection is the cornerstone of management, with the laparoscopic approach considered feasible and safe. Four out of 10 patients might need more than 3 days of hospitalization. In the presence of mass, the frozen section can help guide the management.

## INTRODUCTION

Xanthogranulomatous cholecystitis (XGC) is a rare condition, initially identified by Christensen and Ishak in 1970 and later referred to as XGC by McCoy *et al*. in 1976. It is characterized by a destructive inflammatory reaction that can be focal or diffuse [1]. The chronic inflammation caused by XGC is marked by the production of xanthogranulomas, extensive proliferative fibrosis, and the infiltration of macrophages and foam cells into the gallbladder wall [2]. Although the etiology is unknown, XGC is commonly associated with gallstones and cholestasis. XGC is caused by an inflammatory and granulomatous response, followed by bile extravasation into the gallbladder wall [3]. Consequently, acute inflammation is accompanied by a granulomatous reaction and a cellular immune response [4]. XGC can be severe, extending into adjacent tissues such as the liver, bowel and stomach, causing adhesions, perforation, abscess and fistulous connection with the surrounding bowel [5].

Further, the gallbladder wall thickening and a fibrous response result in gross adhesions, complicating surgical dissection [6, 7]. As a result, it is regularly misdiagnosed as gallbladder carcinoma by ultrasound and CT imaging. [8]. The diagnosis is confirmed by a postoperative histological examination [9]. The current gold standard for treating benign gallbladder disease is laparoscopic cholecystectomy; nevertheless, XGC is linked to a high conversion rate to open. [1]. Preoperative distinctions between XGC and carcinoma must be addressed to avoid severe morbidity, particularly in radical surgery. There is a need to gather evidence in the literature about XGC; additionally, no article published in Saudi Arabia discusses such peculiar pathology.

## CASE SERIES

**Figure 1:**
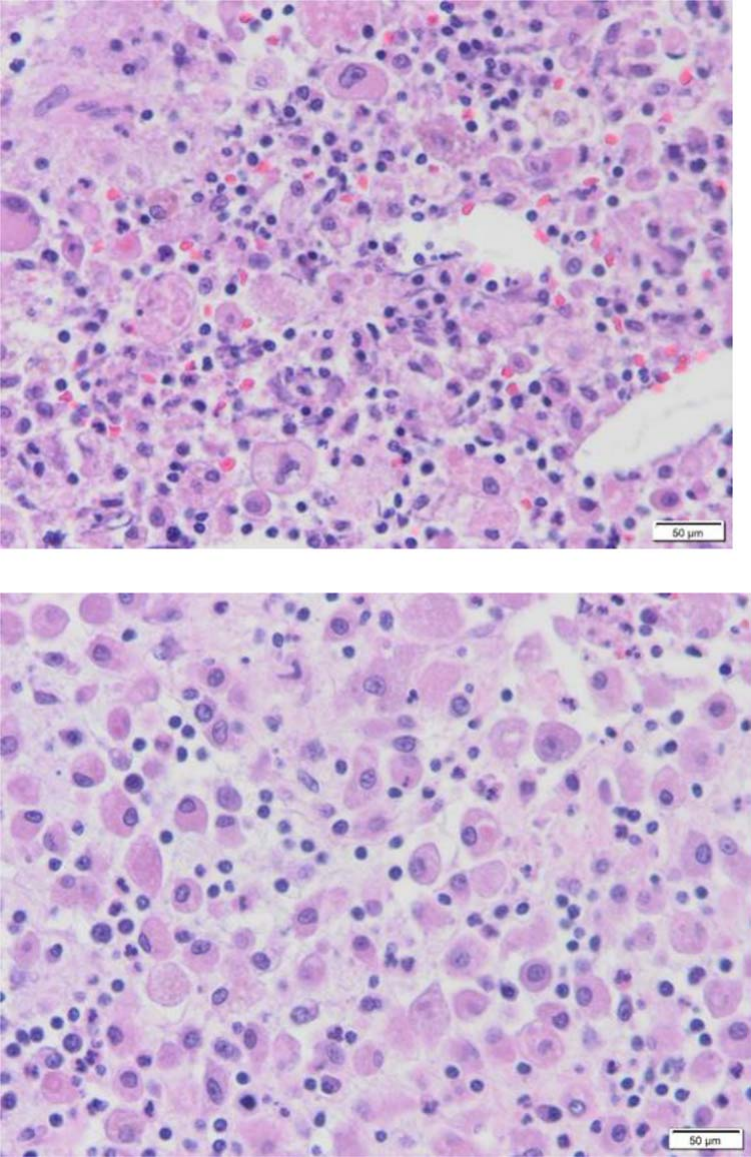
Diffuse proliferation of histiocytes with foreign body giant cell granuloma, Hematoxylin and Eosin H&E, 40x high power filed.

The patient evaluation and the data collection of gallbladder disease were prospectively performed at a single, private tertiary center, Dr Soliman Fakeeh Hospital (Jeddah, Saudi Arabia). The current study investigated the incidence of XGC in 1141 consecutive patients who underwent cholecystectomy in our institution between January 2019 and December 2021. We excluded pediatric age groups and patients who did cholecystectomy combined with other surgeries. The gallbladder disease was confirmed by ultrasonography (US), computed tomography (CT), magnetic resonance cholangiopancreatography (MRCP) and diagnostic laparoscopy. The measured outcome included surgical intervention, conversion to open and complication rate. Factors that might extend the length of hospital stay postoperatively were also evaluated. The Institutional Review Board approved the study protocol of Dr. Soliman Fakeeh Hospital with approval no. 228/IRB/2021. Of 1141 patients who underwent cholecystectomy, XGC was seen in 10 (0.87%). The average age was 47 (24–81 years). Six patients were male, and four were female, with a male-to-female ratio of 3:2. The patients’ nationalities varied, including Arab (8/10), Filipino, and Irish. Comorbidity was present in five patients. The average body mass index (BMI) was 30.8 (27.7–34.5). The duration of symptoms ranged between 1 and 12 weeks. The preoperative diagnoses were biliary colic (5/10), acute cholecystitis (3/10), biliary pancreatitis and obstructive jaundice. Endoscopic retrograde pancreaticography (ERCP) and stenting were done on two patients preoperatively. The average WBC was 8.3 x 10 ^3^ (3.94–15.52), total bilirubin was 0.71 mg/dl (0.18–1.81) and international normalized ratio (INR) was 0.9 (1.2–0.98). The preoperative ultrasonography did not predict the presence of XGC or suggest the need for further imaging, except in one patient with soft-tissue mass inside the gallbladder. The average wall thickness was 5 mm (2–37), stone size was 7 mm [5–13], CBD diameter was 4.5 mm [2–7] and pericholecystic fluid was present only in 20% (2/10). One patient was described to have a mural diverticula and one pyocele. Four out of ten (40%) required more than 72 h of hospitalization. All patients underwent elective surgery with a laparoscopic approach. One patient required conversion to open due to a thickened 12 mm wall with perforation. The average operative time was 90 min (43–193), with no significant intraoperative event. The drain was inserted in 40% of the patients (4/10). One patient required excision of the liver bed; this section was frozen immediately and returned as negative for malignancy. No postoperative complications were listed over the median follow-up of 24 months (11–30). None of the patients had a postoperative collection, bleeding, surgical site infection, biliary leak or readmission. Tables 1 and 2 summarized the patients data. One patient was diagnosed preoperatively on images as suspicious of XGC versus malignancy. The characteristics shared histological features, including ulcerated mucosa with transmural acute and chronic inflammatory cell infiltrate, a sheet of foamy histocyte and multinucleated foreign body giant cell granuloma (Fig. 1).

**Table 1:** Patient demographic and surgical data

Patient	Age	Gender	Diagnosis	Sitting	Conversion	Complication	Drain	Hospital stay (days)
1	81	Male	Biliary pancreatitis	Elective	No	No	No	2
2	47	Female	Biliary colic	Elective	No	No	No	3
3	60	Male	Acute cholecystitis	Elective	Yes	No	Yes	5
4	39	Male	Acute cholecystitis	Elective	No	No	No	2
5	24	Female	Acute cholecystitis	Elective	No	No	No	2
6	38	Male	Biliary colic	Elective	No	No	Yes	2
7	38	Male	Obstructive jaundice	Elective	No	No	No	2
8	36	Female	Biliary colic	Elective	No	No	Yes	3
9	60	Female	Biliary colic	Elective	No	No	Yes	4
10	61	Male	Chronic cholecystitis	Elective	No	No	No	3

**Table 2:** Patient radiological and pathological data

Patient	Symptoms duration (Weeks)	White blood count	Wall thickness (mm)	Average stone size (mm)	US finding	Pathology finding
1	2	5.77	6	7	Pericholecystic fluid	Ulcerated mucosa, foamy histocyte
2	1	11.07	4	5	Multiple stones	Foamy histocyte and multinucleated giant cells
3	1	12.16	12	10	Mural diverticula	Chronic xanthogranulomatous cells
4	12	6.96	3	3	Biliary mud	Ulcerated mucosa, foamy histocyte
5	8	8.88	4	10	Mucocele	Foamy macrophages and cholesterol granuloma
6	2	5.86	6	5	Multiple stones	Ulcerated mucosa, foamy histocyte, and giant foreign body
7	8	6.90	7	13	Wall edema	Foreign body giant cell with a sheet of histocytes
8	4	15.52	2	5	Multiple stones	Transmural extensive macrophages infiltrate
9	2	6.12	2	5	Multiple stones	Foreign body giant cell granuloma with ulcerated mucosa
10	12	3.94	4	Mud	4 cm mass	Severe necrotizing granuloma with chronic cholecystitis, xanthogranulomatous, negative for malignancy, tuberculosis, lymphoma, or parasite

## DISCUSSION

XGC is a rare inflammatory chronic gallbladder disorder that accounts for 0.7–13.2% of all cholecystitis cases [1, 3, 6]. In our review, 10 cases were diagnosed with XGC out of 1441 (0.87%). Our sample was limited to people aged 24–81 years, with an average age of 47 years, consistent with the article suggesting that age may be a risk factor for XGC [11].

In previous studies, XGC was more frequently seen in men than women [8], the same in our sample, with a 3:2 male-to-female ratio. Furthermore, research has shown that male-to-female ratios vary, with some studies indicating that males dominate—such as Guzman-Valdivia’s report (M:F = 107:54) and Han and Chen’s report (M:F = 30:9)—while others, like Youcel *et al*., conclude that XGC affects both men and women equally [1]. These varying reports reflect the low incidence rate of XGC and the missing extensive clinical data, emphasizing the need for larger studies investigating the disease. Although sudden weight loss has been documented as a clinical presentation of XGC [12], our cases presented a high BMI of 30.8.

XGC is generally physiologically aggressive, spreading through the gallbladder wall into neighboring tissues and causing substantial morbidity [12, 13]. As a result, XGC is thought to have a unique clinical feature and an invasive form of chronic cholecystitis [13]. Gallbladder stones and biliary obstruction may contribute to the development of XGC by allowing bile to extravasate into the gallbladder wall through ruptured Rokitansky-Aschoff sinuses and mucosal surface ulcers [4]. The researchers have indicated a significant correlation between XGC and cholelithiasis; all the patients in our study had a 7 mm [5–13] gallbladder stone, supporting that cholelithiasis has a role in the development of XGC [16].

Generally, clinical manifestations of XGC are not specific and are confused with other illnesses, such as gallbladder carcinoma, and commonly present as abdominal pain, especially in the upper quadrant, jaundice, nausea and vomiting [1, 9]. In the present report, approximately half of the patients (5/10) experienced biliary colic, which cannot help diagnose XGC. Despite the existence of clinical, radiological and laboratory evidence, distinguishing XGC from gallbladder cancer remains challenging [14–16]; histopathology remains the gold standard [17, 18].

Makimoto *et al*. [13] indicated that 26 (83.9%) cases were diagnosed as suspicious XGC using preoperative ultrasonography and computed tomography findings. Conversely, only one case was detected as XGC perioperatively on images in our sample 10% (1/10). While the data were consistent with XGC reported in cross-sectional imaging retrospective analysis, we believe that radiologists and clinicians are still unaware of this rare condition.

Surgery is the only effective therapy for XGC [19]. Such findings direct treatment choice depending on the thickness of the wall, malignancy, involvement of adjacent structures and the need for radical surgery, which also impact the duration of the procedure, complication rate and hospital stay [20]. Patients with XGC had a greater risk of conversion to open surgery than patients with other types of cholecystitis, with rates ranging from 19 to 80% [21]. In contrast to these findings, only 1 patient out of 10 (10%) required a conversion in our review due to a thickened 12 mm wall with perforation. Two patients underwent prior ERCP and stent procedures due to gallbladder stones, while only one had a mural diverticula and one pyocele.

Although biliary injury, bile leakage, bleeding and surgical site infection are all possible postoperative complications [6], none of the patients in our study suffered any. However, approximately four patients required more than 72 h in the hospital (an average of 2.8 days), which is less than a recent large-case series reported by Youcel *et al*. [1], where the average hospital stay was 5.1 days.

## CONCLUSIONS

XGC is a rare benign entity requiring no further action upon incidental discovery. Surgical resection is the cornerstone in the management, with the laparoscopic approach considered feasible and safe. The rate of intraoperative events and postoperative complications are comparable with non-XGC. The average hospital stay is 3 days, and the surgical drain might be needed in half of the patients due to difficult dissection. An intraoperative frozen section can help differentiate XGC from cancer.

## AUTHORSHIPS AND CONTRIBUTIONS

A.M.A. contributed to study conception and design, data acquisition, analysis, interpretation, drafted a critical manuscript revision and approved the text’s final version. E.A. contributed to study conception and design, data acquisition, analysis, and interpretation drafted a critical manuscript revision, and approved the text’s final version. H.A. contributed to study conception and design, data acquisition, analysis, and interpretation, drafted a critical manuscript revision, and approved the text’s final version. A.A. contributed to study conception and design, data acquisition, analysis, interpretation, drafted a critical manuscript revision, and approved the text’s final version.

## COMPLIANCE WITH ETHICAL STANDARDS

Disclosure: Abdulrahman Alotaibi, Eid Almasoudi, Hassan Ahmed, and Abubakr Alzwaihiri, have no conflict of interest or financial ties to disclose.
